# MicroRNA-125a-5p Mediates 3T3-L1 Preadipocyte Proliferation and Differentiation

**DOI:** 10.3390/molecules23020317

**Published:** 2018-02-02

**Authors:** Yan Xu, Jingjing Du, Peiwen Zhang, Xue Zhao, Qiang Li, Anan Jiang, Dongmei Jiang, Guoqing Tang, Yanzhi Jiang, Jinyong Wang, Xuewei Li, Shunhua Zhang, Li Zhu

**Affiliations:** 1 College of Animal Science and Technology, Sichuan Agricultural University, Chengdu 611130, China; xuyanyan0924@sina.com (Y.X.); 18180008728@163.com (J.D.); sicau_zhangpeiwen@163.com (P.Z.); 18227588896@163.com (X.Z.); 13540384633@139.com (A.J.); jiangdm@sicau.edu.cn (D.J.); tyq003@163.com (G.T.); xuewei.li@sicau.edu.cn (X.L.); 2 Sichuan Province General Station of Animal Husbandry, Chengdu 611130, China; 18398635864@163.com; 3 College of Life and Science, Sichuan Agricultural University, Chengdu 611130, China; jiangyz04@163.com; 4 Chongqing Academy of Animal Sciences, Chongqing 402460, China; kingyou@vip.sina.com

**Keywords:** miR-125a-5p, STAT3, preadipocyte, 3T3-L1, obesity

## Abstract

Excessive accumulation of adipose tissue is a main cause of obesity or overweight, which is significantly involved in increasing the risk of diseases. Recently, numerous studies have proved that microRNAs (miRNAs) play important roles in adipogenesis by negatively regulating gene expression at posttranscriptional levels. In this study, we showed that miR-125a-5p was expressed at lower levels in the adipose tissues of high-fat diet (HFD)-fed mice than the normal chow (NCW)-fed mice. MiR-125a-5p expression were strongly up-regulated by nearly five-fold, when 3T3-L1 preadipocyte were induced and differentiated into mature adipocytes. Functional analysis indicated that overexpression of miR-125a-5p promoted 3T3-L1 preadipocyte proliferation and inhibited its differentiation. By contrast, inhibition of miR-125a-5p repressed 3T3-L1 preadipocyte proliferation and accelerated its differentiation. Furthermore, a dual-luciferase reporter assay demonstrated that signal transducer and activator of transcription 3 (STAT3) is a direct target gene of miR-125a-5p during 3T3-L1 preadipocyte differentiation. Further analysis confirmed that the process of miR-125a-5p inhibiting 3T3-L1 preadipocyte differentiation might be associated with the regulation of fatty acid metabolism related genes. Taken together, our results indicated that miR-125a-5p might promote 3T3-L1 preadipocyte proliferation, whereas inhibiting 3T3-L1 preadipocyte differentiation by negatively regulating STAT3.

## 1. Introduction

In the past few decades, obesity or overweight were usually considered a major nutritional health problem in developed countries [1]. Now, the prevalence of obesity is significantly increasing on a world scale, which has reached epidemic proportions and become among the most common human health problems in developed and developing countries [2,3]. For example, nearly half a billion of the world’s population are now considered to be overweight or obese [4]. Particularly, the proportion of adults with a body-mass index (BMI) of 25 kg/m^2^ was greatly increased between 1980 and 2013 from 28.8% to 36.9% in men, and from 29.8% to 38.0% in women [5]. Notably, overweight and obesity were estimated to cause 3.4 million deaths in 2010 [5], and increasing evidence indicates that obesity or overweight might be a main risk factor of several chronic diseases, such as nonalcoholic fatty liver disease, cardiovascular disease, hypertension type 2 diabetes and cancers [6,7,8]. Therefore, limiting overweight and treating obesity are important for treating obesity-related diseases and improving human health.

However, the reasons for obesity or overweight formation are complicated, which might be associated with genetic and environmental influences. Previously, many studies have indicated that disruption in energy balance leading to excessive accumulation in adipose tissue is a major manifestation of obesity or overweight [2,9,10]. Therefore, completely understanding the molecular mechanism of adipocytes development is critical for limiting obesity and treating obesity-related diseases. MicroRNAs (miRNAs) are endogenous 18–22 nt and play important roles in various biological process, including cell proliferation, differentiation, death and apoptosis [11,12,13,14]. Recently, numerous studies demonstrated that miRNAs are key for the regulation of adipogenesis. For instance, miR-155 could mediate differentiation of brown and beige adipocytes via a bistable circuit [15]. MiR-125a-3p and miR-483-5p promoted adipogenesis by suppressing the RhoA/ROCK1/ERK1/2 pathway in multiple symmetric lipomatosis [16]. Additionally, Cioffi et al. [17] showed that as a negative regulator of adipogenesis, miR-93 might be a potential therapeutic option for obesity and the metabolic syndrome. In the present study, we found that expression levels of miR-125a-5p in adipose tissues was negatively correlative with HFD-induced obesity. By transfecting miR-125a-5p mimics, inhibitors or the negative control into 3T3-L1 preadipocyte, we showed that miR-125a-5p promoted 3T3-L1 preadipocyte proliferation via affecting cell cycle-regulated factors, whereas inhibited differentiation through directly targeting STAT3, and this effect might be associated with fatty acids oxidation and synthesis.

## 2. Materials and Methods

### 2.1. Experimental Animals

As described by our previous study [18], two groups of six-week-old Kunming mice (male) were fed a high-fat diet (HFD) or normal chow (NCW) for 15 weeks. During the experiment, mice were given free access to water, HFD or NCW under controlled conditions of light and temperature. In the end, body weight and serum levels of total cholesterol (TC) and triglycerides (TG) were measured, and adipose tissues were collected and stored at −80 °C for further analysis. All experimental mice were carried out in accordance with the U.K. Animals (Scientific Procedures) Act, 1986 and associated guidelines (Approval document: DKYS20163643).

### 2.2. Cell Culture and Transfection

Before differentiation, mouse 3T3-L1 preadipocyte (Stem Cell Bank, Chinese Academy of Science) were maintained in proliferation medium containing Dulbecco’s modified Eagle’s medium (DMEM, Gibco, Carlsbad, CA, USA) supplemented with 10% fetal bovine serum (FBS) at 37 °C with 5% CO_2_. In order to induce differentiation, proliferating cells were stimulated firstly in differentiation medium for two days, containing 10% FBS, 10 μg/mL insulin, 1 μM dexamethasone, and 0.5 mM IBMX, and then the media was changed to maintenance media containing 10% FBS and 10 μg/mL insulin for two days. Finally, cells were maintained in DMEM containing 10% FBS until 8th day of differentiation.

Furthermore, to explore the effect of miR-125a-5p on 3T3-L1 preadipocyte proliferation and differentiation, using Lipofectamine2000 (Invitrogen, Carlsbad, CA, USA), cells were respectively transfected with synthetic miR-125a-5p mimics (50 nM, catalog number: miR10000135-1-5), inhibitors (50 nM, catalog number: miR20000135-1-5) or negative control (50 nM) (all purchased from RiboBio, Guangzhou, China), according to the manufacturer’s instructions.

### 2.3. Quantitative RT-PCR

According to the manufacturer’s instructions, total RNA from cells and adipose tissues were extracted using TRIzol Reagent (Invitrogen, Carlsbad, CA, USA). Subsequently, reverse transcription of mRNA and miRNA was performed using a commercial kit (TaKaRa, Chengdu, China), following the manufacturer’s protocol. Quantitative PCR was performed using the SYBR Premix Ex Taq kit (TaKaRa, Chengdu, China) on a CFX96 system (Bio-Rad, Berkeley, CA, USA). Relative expression levels of mRNAs and microRNAs were calculated using the 2^−ΔΔCt^ method [19]. The primer sequences used for qRT-PCR are listed in Supplementary Table S1.

### 2.4. CCK-8 and EdU Assays

Briefly, proliferation of 3T3-L1 preadipocyte transfected respectively with mi125a-5p mimics, inhibitors or negative control were measured at 0 h, 12 h, 24 h, 48 h and 72 h using a Cell Counting kit 8 (CCK-8, Beyotime, Shanghai, China), according to a previous report as described by Du et al. [18]. For 5-ethynyl-2′-deoxyuridine (EdU) proliferation analysis, cells were firstly treated with 10 μM EdU (RiboBio, Guangzhou, China) for 24 h, then incubated for a further 12 h. Subsequently, EdU staining was performed according to the manufacturer’s protocol. Images were captured using an Olympus IX53 microscope (Olympus, Tokyo, Japan).

### 2.5. Immunocytochemical Analysis

According to previous report by Du et al. [18], when differentiating adipocytes were transfected with mi-125a-5p mimics, inhibitors or negative control for 8 days, immunocytochemical analysis was performed. Briefly, cells were washed 3 times with phosphate-buffered saline (PBS) and fixed in 4% paraformaldehyde for 15 min. After further PBS washes, the cells were then permeabilized with 0.5% Trixon X-100 prior to blocking in 2% goat serum (diluted in PBS). After blocking, cells were incubated with an anti-adiponectin antibody at 37 °C for 2 h then fluorescent secondary antibodies at 37 °C for 1 h. The nuclei were stained with 4′,6-diamidino-2-phenylindole (DAPI, Boster, Wuhan, China) for 10 min. Images were captured using an Olympus IX53 microscope (Olympus, Tokyo, Japan).

### 2.6. Oil Red O Staining and Triglyceride Assay

Briefly, cells were washed with PBS, and fixed in 4% paraformaldehyde for 120 min. Then, fixed cells were stained with 0.5% Oil Red O for 2 h at room temperature. Subsequently, after cells were washed 3 times with PBS, images were captured using an Olympus IX53 microscope (Olympus, Tokyo, Japan). For triglyceride assay, stained cells were eluted with isopropanol for 20 min, and the OD values were read at 510 nm wavelength.

### 2.7. Luciferase Reporter Assay

The wild-type 3′ UTR of STAT3 (WT-STAT3), Mutant-type 3′ UTR of STAT3 (Mut-STAT3) were made by a manufacturer (TsingKe Biotech, Chengdu, China). For the luciferase reporter analysis, using Lipofectamine2000 (Invitrogen), miR-125a-5p mimics, inhibitors or NC were co-transfected with the wild-type 3′ UTR or mutant-type 3′ UTR into 3T3-L1 cells (Stem Cell Bank, Chinese Academy of Science). Cells were harvested 48 h after transfection, and luciferase activities were measured with the Dual-Glo Luciferase Assay System (Promega, Madison, WI, USA) following the manufacturer’s instructions. Additionally, firefly luciferase was used as a normalization control.

### 2.8. Statistical Analysis

Data are presented as means ± SE. Using SPSS 22.0, the statistical significance between groups was determined with student’s *t*-test, ANOVA were performed to compare means between more than 2 groups. A value of *p* < 0.05 indicated a significant difference.

## 3. Results and Discussion

### 3.1. MiR-125a-5p Is Involved in Adipogenesis

**Figure 1:**
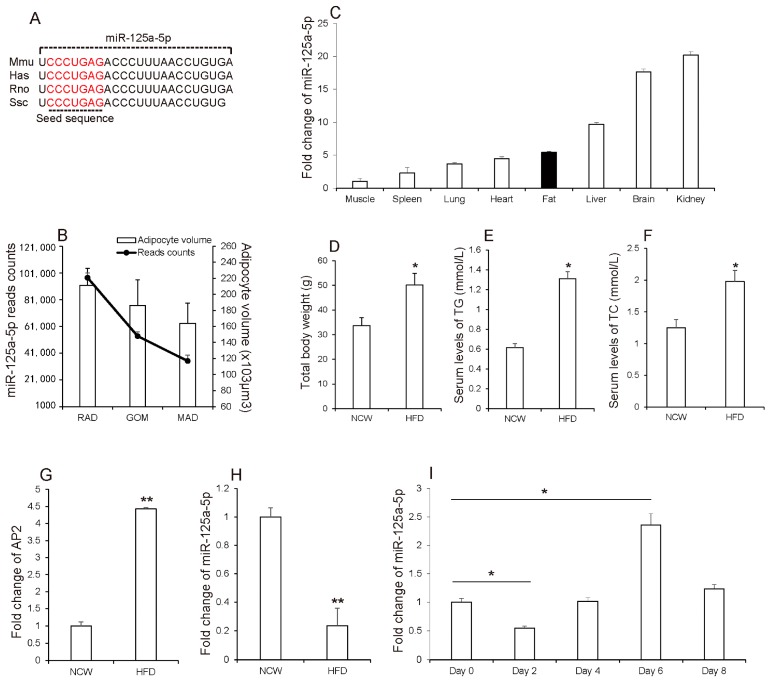
miR-125a-5p is associated with adipogenesis. (**A**) Mature sequence of miR-125a-5p was conserved among species including mouse (Mmu), human (Hsa), Rat (Rno), swine (Ssc); (**B**) the expression of miRNA-125a-5p using Illumina Genome Analyzer II and adipocyte volume in different adipose tissues of swine, including retroperitoneal adipose (RAD), greater omentum (GOM) and mesenteric adipose (MAD); (**C**) the expression of miR-125a-5p in different tissues of mice. Additionally, after kunming mice were fed high-fat diet (HFD) or normal chow (NCW) diet, (**D**) body weight, (**E**,**F**) serum levels of TG and TC and the mRNA levels of (**G**) AP2 and (**H**) miR-125a-5p were measured; (**I**) The expression levels of miR-125a-5p during 3T3-L1 preadipocyte differentiation. Results are presented as means ± SEM. *n* = 3. * *p* < 0.05; ** *p* < 0.01.

Mature miR-125a-5p sequence is highly homologous in various species including human, mouse, rat and swine (Figure 1A). Previously, our study has shown that ssc-miR-125a-5p was expressed at lower levels in adipose tissues containing large volumes of adipocytes, of which increasing retroperitoneal adipose (RAD), greater omentum (GOM) and mesenteric adipose (MAD) have been demonstrated to be associated with several diseases [20] (Figure 1B), and Ji et al. [21] demonstrated that ssc-miR-125a-5p inhibited porcine preadipocyte differentiation by targeting ERRα. Here, qRT-PCR analysis showed that mmu-miR-125a-5p has a considerable level of expression in the adipose tissue of mice (Figure 1C). The main proponent of weight gain during obesity is white adipose tissue (WAT). To further investigate the relationship between miR-125a-5p and adipocyte development, Kunming mice were fed a high-fat diet (HFD) to induce obesity. As shown in (Figure 1D–F), HFD feeding significantly increased body weight and serum levels of total TC and TG when compared to normal chow (NCW) feeding, which has been demonstrated by Tan et al. [22]. These results suggested that mice got obesity under HFD. Consistently, in contrast to that AP2, a marker of obesity and adipocyte [23,24], which highly expressed in adipose tissues of HFD-fed mice, miR-125a-5p was expressed at lower levels in adipose tissues of HFD-fed mice than NCW-fed mice (Figure 1G,H), suggesting that expression levels of miR-125a-5p in adipose tissues might be negatively correlated with HFD-induced obesity. Subsequently, to confirm the relationship between miR-125a-5p and adipogenesis, we evaluated the expression levels of miR-125a-5p during 3T3-L1 preadipocyte differentiation into mature adipocytes, which is widely used as a model of adipogenesis [25]. As shown in Figure 1I, we found that expression levels of miR-125a-5p in proliferating cells (day 0) was strongly decreased after adipocytes were induced into differentiation for two days, and then gradually increased by nearly five-fold up to day 6 of adipocyte differentiation. Interestingly, miR-125a-5p expression in day 8 of adipocyte differentiation was remarkably decreased by 50%, when compared to that of day 6. This data showed a similar expression pattern with miR-224, which have been demonstrated to impair adipocyte early differentiation [26]. Considering these findings, we thus speculated that miR-125a-5p might be involved in adipogenesis.

### 3.2. MiR-125a-5p Promotes 3T3-L1 Preadipocyte Proliferation

**Figure 2:**
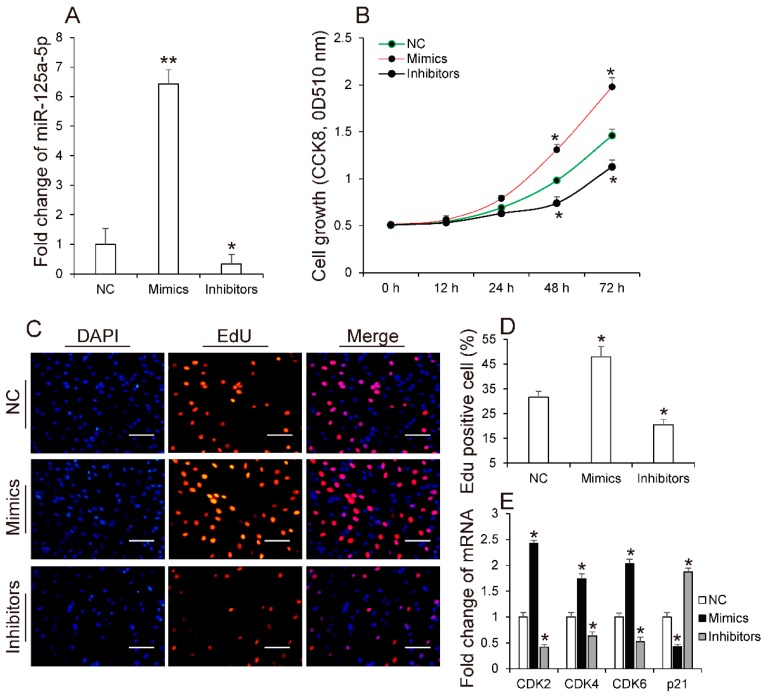
miR-125a-5p promotes 3T3-L1 preadipocyte proliferation. After 3T3-L1 preadipocyte were transfected, respectively, with miR-125a-5p mimics, inhibitors or negative control (NC), (**A**) the expression levels of miR-125a-5p were measured by performing qRT-PCR; (**B**) cells proliferation were evaluated by CCK8 (**C**,**D**) EdU analysis, respectively. Moreover; (**E**) the mRNA levels of CDK2, CDK4, CDK6 and p21 were measured by qRT-PCR. Scale bar, 10 μm. Results are presented as means ± SEM. *n* = 3. * *p* < 0.05; ** *p* < 0.01.

It is known that adipocyte proliferation and differentiation are the basis for the accumulation of lipids in adipose tissues [27]. To identify the role of miR-125a-5p in adipogenesis, we first explored whether miR-125a-5p affects adipocyte proliferation by respectively transfecting miR-125a-5p mimics, inhibitors or negative control into 3T3-L1 preadipocyte. As shown in (Figure 2A), transfection of mimics significantly increased the expression levels of miR-125a-5p in 3T3-L1 preadipocyte, when compared to the negative control group (NC). In contrast, endogenic expression level of miR-125a-5p was remarkably decreased in cells transfected with inhibitors. Subsequently, EdU and CCK8 analysis were performed to evaluate the effect of miR-125a-5p on adipocyte proliferation. As shown in (Figure 2B), CCK8 analysis indicated that when compared to NC, the total number of 3T3-L1 preadipocytes was significantly increased or decreased in mimics or inhibitor groups, respectively. This finding was also demonstrated by EdU analysis. EdU detection showed that overexpression of miR-125a-5p increased the ratio of EdU-positive cells, when compared to NC. In contrast, inhibition of miR-125a-5p decreased the ratio of EdU-positive cells compared to NC (Figure 2C,D). Cyclin-dependent kinases such as CDK2, CDK4 and CDK6 play key roles in positively regulating the cell cycle, which are required for the G1/S and G2/M transitions [28]. Conversely, p21 has been identified as a cell cycle-arrest regulator [29]. In order to validate whether miR-125a-5p mediates 3T3-L1 preadipocyte proliferation and is associated with CDKs and p21, we subsequently measured the expression levels of these factors. Consistent with the above observation, qRT-PCR analysis indicated that overexpression of miR-125a-5p remarkably enhanced expression of CDK2, CDK4 and CDK6, and inhibition of miR-125a-5p suppressed the expression of these genes, when compared to NC (Figure 2E). Conversely, when compared to NC, the expression of p21 was decreased or increased in cells transfected with mimics or inhibitors, respectively (Figure 2E). Previously, several studies have indicated that miRNAs promoting or inhibiting preadipocyte proliferation are usually much lower or higher in HFD-induced obese mice than NCW-fed mice, respectively [22,30]. This finding that miR-125a-5p promotes 3T3-L1 preadipocyte proliferation is also consisted with an above result that miR-125a-5p is expressed at lower levels in adipose tissues of HFD-fed mice than NCW-fed mice. Taken together, these results collectively suggested that miR-125a-5p might promote 3T3-L1 preadipocyte proliferation.

### 3.3. MiR-125a-5p Inhibits 3T3-L1 Preadipocyte Differentiation by Targeting STAT3

**Figure 3:**
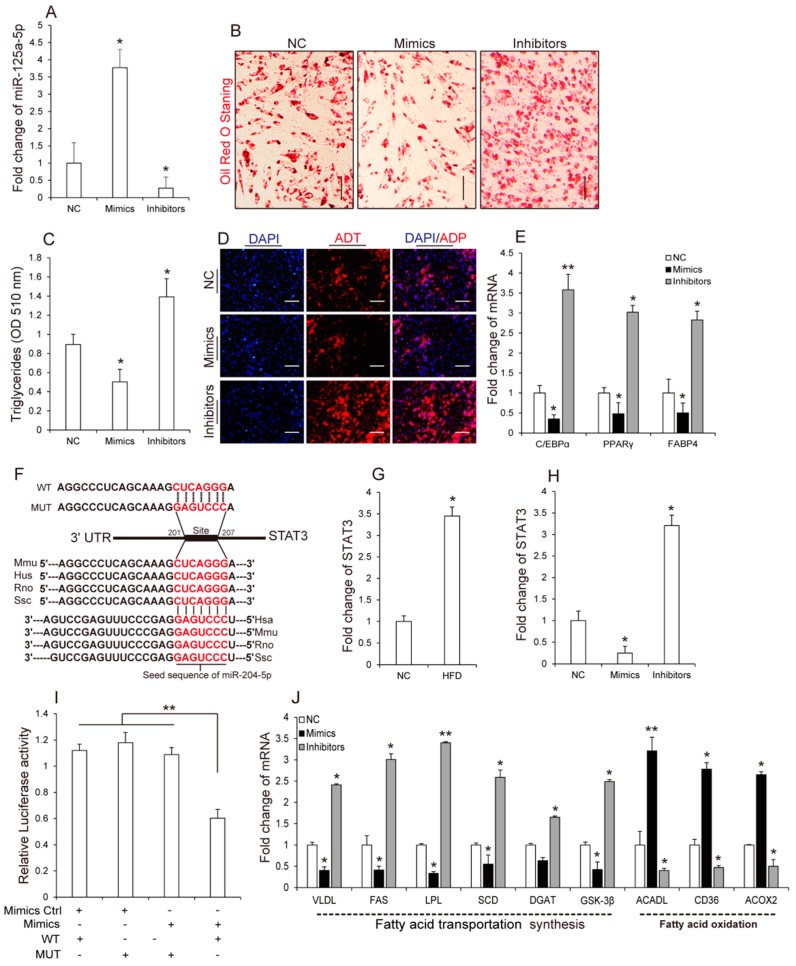
miR-125a-5p inhibits 3T3-L1 preadipocyte differentiation. After differentiation, cells were transfected with miR-125a-5p mimics, inhibitors or NC for eight days. (**A**) The expression levels of miR-125a-5p were measured by qRT-PCR; (**B**) Oil Red O staining of terminally differentiated adipocytes; (**C**) the content of triglycerides in terminally differentiated adipocytes; (**D**) the expression of adiponectin was evaluated by performing immunofluorescence analysis; (**E**) the mRNA levels of C/EBPα, PPARγ and FABP4 were measured by qRT-PCR; (**F**) sequence alignment of miR-125a-5p with 3′ UTR of Mmu, Hsa, Rno and Ssc STAT3 mRNA. Binding site and seed region of miR-125a-5p are indicated in red. Additionally, the recombinant double fluorescent reporter plasmid contains either a wild-type (WT) 3′ UTR of STAT3 or mutant (WT) 3′ UTR of STAT3, respectively; (**G**) The expression levels of STAT3 in adipose tissues from HFD- or NCW-fed mice; (**H**) The expression levels of STAT3 in cells transfected with miR-125a-5p mimics, inhibitors or negative control (NC); (**I**) Luciferase assays revealed the repressive effect of miR-125a-5p on the activity of STAT3; (**J**) The expression levels of genes related to fatty acid oxidation and fatty acid synthesis in differentiated cells transfected with miR-125a-5p mimics, inhibitors, NC. Scale bar, 10 μm. Results are presented as means ± SEM. *n* = 3. * *p* < 0.05; ** *p* < 0.01.

Next, we investigated the effect of miR-125a-5p on adipocyte differentiation. As shown in (Figure 3A), overexpression of miR-125a-5p significantly inhibited 3T3-L1 preadipocyte differentiation as determined by analyzing Oil Red O staining signals (Figure 3B) and triglycerides (TG) accumulation (Figure 3C) and adiponectin (Figure 3D) using immunofluorescence at day 8 of differentiation. Moreover, in accordance with these findings, we found that transfection of miR-125a-5p mimics or inhibitors in 3T3-L1 preadipocytes significantly inhibited or enhanced adipogenic transcripts (C/EBPα, PPARγ and FABP4) (Figure 3E), respectively. These results suggested that miR-125a-5p might inhibit 3T3-L1 preadipocyte differentiation. Previously, several studies have proven that miRNAs take part in various biological processes by negatively regulating their target genes [31,32,33,34]. To further identify potential molecular mechanisms by which miR-125a-5p inhibited 3T3-L1 preadipocyte differentiation, using TargetScan, we predicted potential target genes of miR-125a-5p. Among the predicted target genes, signal transducer and activator of transcription 3 (STAT3) contains a complementary sequence to miR-125a-5p in the 3′ UTR region (Figure 3F) and previous studies have demonstrated that STAT3 is necessary for 3T3-L1 adipocyte formation, which can accelerate differentiation of 3T3-L1 preadipocytes [35,36]. Additionally, in contrast to the miR-125a-5p that has a lower expression in adipose tissue of HFD-fed mice, STAT3 has a higher expression in adipose tissues of HFD-fed mice, when compared to that of NCW-fed mice (Figure 3G). Overexpression of miR-125a-5p significantly inhibited expression levels of STAT3, whereas inhibition of miR-125a-5p strongly increased STAT3 expression during adipocyte differentiation (Figure 3H), suggesting that miR-125a-5p is negatively associated with STAT3. Subsequently, in order to confirm whether miR-125a-5p negatively regulates STAT3 by directly binding to the 3′ UTR region of STAT3 during adipogenesis, a dual-luciferase reporter assay was performed. As shown in (Figure 3I), when compared with the 3T3-L1 cells co-transfected with NC and WT-STAT3, the relative luciferase activity of the 3T3-L1 cells co-transfected with miR-125a-5p mimics and psiCHECK-PTEN wild type (WT-STAT3) was significantly inhibited, whereas mutation of the miR-125a-5p binding site in porcine STAT3 3′ UTR completely abolished this response. Overall, these results suggest that miR-125a-5p might negatively regulate 3T3-L1 preadipocyte differentiation through directly targeting STAT3.

Fatty acids are important components of adipose tissues that increases fatty acid synthesis, however, decreasing fatty acid oxidation can enhance lipid accumulation in adipocytes [37,38]. Interestingly, previous studies have reported that STAT3 could regulate fatty acids to mediate autophagy and cell viability [39,40], thus giving a hypothesis that miR-125a-5p might inhibit 3T3-L1 preadipocyte differentiation by regulating fatty acid metabolism. For this hypothesis, we performed qRT-PCR and measured the expression level of genes related to fatty acid metabolism including fatty acid synthesis and fatty acid oxidation, after cells were transfected with miR-125a-5p mimics, inhibitors or negative control, respectively (Figure 3J). Expectantly, overexpression of miR-125a-5p significantly promoted the expression of genes associated with fatty acid oxidation and repressed the expression of genes involved in fatty acid synthesis, when compared to the control group. In contrast, inhibition of miR-125a-5p had an opposite effect on genes related fatty acid metabolism compared to that of miR-125a-5p overexpression, suggesting that miR-125a-5p could inhibit fatty acid synthesis but promote fatty acid oxidation during 3T3-L1 preadipocyte differentiation. Taken together, these results indicated that miR-125a-5p negatively regulates 3T3-L1 preadipocyte differentiation by directly targeting STAT3, and this effect might be associated with fatty acid metabolism.

## 4. Conclusions

In the present study, miR-125a-5p was found to be highly expressed in adipose tissues of NCW-fed mice, and gradually increased during 3T3-L1 preadiopocte differentiation. Functional analysis showed that miR-125a-5p promoted 3T3-L1 preadipocyte proliferation, and inhibited differentiation by directly targeting STAT3. Furthermore, miR-125a-5p was shown to up-regulate genes associated with fatty acid oxidation, and down-regulate genes involved in fatty acid synthesis. These results confirmed that the expression level of miR-125a-5p in adipose tissues has a negative correlation with obesity in mice and plays an important role in adipogenesis. In particular, miR-125a-5p may be possibly considered as a therapeutic target for obesity.

## Supplementary Materials

The Supplementary Materials are available online.
