# Supplementary material for: MicroRNA-125a-5p Mediates 3T3-L1 Preadipocyte Proliferation and Differentiation
Source: Molecules. 2018 Feb 2;23(2):317. doi: 10.3390/molecules23020317 (PMC6017839; doi:10.3390/molecules23020317)
Supplement: Supplementary file 1 [file molecules-23-00317-s001.pdf]

Gene	Primer sequence (5`→3`)
PPARγ	F:CTCCAAGAATACCAAAGTGCGA<br>R:GCCTGATGCTTTATCCCCACA
CDK6	F:TCTGGCCAGCTCTTCATTCA<br>R:CGGCACACAGTAAGCTCTTC
C/EBPα	F:CAAGAACAGCAACGAGTACCG<br>R:GTCACTGGTCAACTCCAGCAC
CDK4	F: GTCAGTTTCTAAGCGGCCTG<br>R: CACGGGTGTTGCGTATGTAG
CDK2	F: CCCTTCCCAAAGCCCTTTTC<br>R: GAAGAGGGGAAGAAGCTGGT
SCD	F:TTCTTGCGATACACTCTGGTGC<br>R:CGGGATTGAATGTTCTTGTCGT
FAS	F:TATCAAGGAGGCCCATTTTGC<br>R:TGTTTCCACTTCTAAACCATGCT
GSK	F:ATGGCAGCAAGGTAACCACAG<br>R:TCTCGGTTCTTAAATCGCTTGTC
DGAT	F:CTGATCCTGAGTAATGCAAGGTT<br>R:TGGATGCAATAATCACGCATGG
VLDL	F:AGACCAATCAGACGAGTCTCTT<br>R:CTGCCGTCCTTGCAGTCAG
LPL	F:TGGCGTAGCAGGAAGTCTGA<br>R:TGCCTCCATTGGGATAAATGTC
FABP4	F: TTTCCTTCAAACTGGGCGTG<br>R: CATTCCACCACCAGCTTGTC
STAT3	F:AATGGAAATTGCCCGGATCG<br>R:TCCTGAAGATGCTGCTCCAA
P21	F:GATGGCTTCGACACCATTCC<br>R:AGACGACACAGGTGAGGAAG
AP2	F: CGATCCCAATGAGCAAGTGG<br>R: TGGGTCAAGCAACTCTGGAT
miR-125a-5p	UCCCUGAGACCCUUUAACCUGUGA
U6	F:CTCGCTTCGGCAGCACA<br>R:AACGCTTCACGAATTTGCGT
β-actin	F:TGGAATCCTGTGGCATC CATGAAAC<br>R:TAAAACGCAGCTCAG TAACAGTCCG

Supplementary Table S1. The primer sequences used for qRT:PCR. F: forward, R: reverse. U6 and *β-actin* were used as endogenous control genes for miRNA and mRNA, respectively.
